# High Human T Cell Leukemia Virus Type-1(HTLV-1) Provirus Load in Patients with HTLV-1 Carriers Complicated with HTLV-1-unrelated disorders

**DOI:** 10.1186/1743-422X-7-81

**Published:** 2010-04-28

**Authors:** Daisuke Sasaki, Yuko Doi, Hiroo Hasegawa, Katsunori Yanagihara, Kunihiro Tsukasaki, Masako Iwanaga, Yasuaki Yamada, Toshiki Watanabe, Shimeru Kamihira

**Affiliations:** 1 Department of Laboratory Medicine, Nagasaki University Graduate School of Biomedical Sciences, Nagasaki, Japan; 2 Department of Hematology, Nagasaki University Graduate School of Biomedical Sciences, Nagasaki, Japan; 3 Graduate School of Frontier Sciences, University of Tokyo, Tokyo, Japan

## Abstract

**Background:**

To address the clinical and virological significance of a high HTLV-1 proviral load (VL) in practical blood samples from asymptomatic and symptomatic carriers, we simultaneously examined VL and clonal expansion status using polymerase chain reaction (PCR) quantification (infected cell % of peripheral mononuclear cells) and Southern blotting hybridization (SBH) methods.

**Results:**

The present study disclosed extremely high VL with highly dense smears with or without oligoclonal bands in SBH. A high VL of 10% or more was observed in 16 (43.2%) of a total of 33 samples (one of 13 asymptomatic carriers, 8 of 12 symptomatic carriers, and 7 of 8 patients with lymphoma-type ATL without circulating ATL cells). In particular, an extremely high VL of 50% or more was limited to symptomatic carriers whose band findings always contained at least dense smears derived from polyclonally expanded cells infected with HTLV-1. Sequential samples revealed that the VL value was synchronized with the presence or absence of dense smears, and declined at the same time as disappearing dense smears. Dense smears transiently emerged at the active stage of the underlying disease. After disappearance of the smears, several clonal bands became visible and were persistently retained, explaining the process by which the clonality of HTLV-1-infected cells is established. The cases with only oligoclonal bands tended to maintain a stable VL of around 20% for a long time. Two of such cases developed ATL 4 and 3.5 years later, suggesting that a high VL with oligoclonal bands may be a predisposing risk to ATL.

**Conclusion:**

The main contributor to extremely high VL seems to be transient emergence of dense smears detected by the sensitivity level of SBH, corresponding to polyclonal expansion of HTLV-1-infected cells including abundant small clones. Major clones retained after disappearance of dense smears stably persist and acquire various malignant characteristics step by step.

## Background

Human T-cell leukemia virus type-1 (HTLV-1) is thought to infect mainly CD4 T-cells, and to cause T-cell malignancy adult T-cell leukemia (ATL) after a long latency, a degenerative nervous disorder of HTLV-1-associated myelopathy (HAM), and so on [1,2]. During the clinically asymptomatic period preceding the diseases, the HTLV-1-infected cell number is low, at about less than 2 - 3% per 100 blood mononuclear cells (MNC). Therefore, infected cells in asymptomatic (healthy) carriers are considered to proliferate polyclonally because the provirus integrates at a random site [3]. Recent work using real-time polymerase chain reaction (PCR) quantification for HTLV-1 provirus (proviral load: VL) and inverse PCR indicates that clonal expansion of HTLV-1-infected cells is important for the maintenance of infection [4-6]. Interestingly, the proviral integration sites in genomic DNA in asymptomatic and symptomatic carriers without ATL is either random or constant, implying the difference in clonality detected by Southern blotting hybridization (SBH) [7,8]. Thus, high VL corresponding to an increased number of polyclonal or monoclonal infected cells is one of the key events in HTLV-1-associated pathology. Therefore, a high VL with clonal expansion has potential as a biomarker to predict patients predisposed to ATL or HAM [9,10]. On the other hand, HTLV-1-infected individuals who are complicated by opportunistic infections, such as parasites, mycosis, viruses and some bacteria, and abnormal immunity due to aging are known to show an increased proviral load with polyclonal expansion [11-13]. This condition associated with polyclonal expansion of the infected cells was considered to be the intermediate state prior to progression to ATL [14], but the pathological and clinical correlation between clonality and level of VL is not fully understood. Recently, we have had frequent opportunities to see unusual or indeterminate ATL patients or carriers with high VL with discrete band(s) in SBH, but no circulating ATL cells, especially among the elderly.

Accordingly, to address what kind of clonal infected cells contribute to high VL, and clarify unusual ATL or carrier states, we simultaneously analyzed HTLV-1 proviral load and SBH status using the same blood samples. In contrast to the maintenance of stable VL in asymptomatic carriers with no-bands or only faint discrete bands, the VL in symptomatic carriers with complications unrelated HTLV-1 tended to have high VL with dense smears with/without discrete band(s), consisting of mainly polyclonal expansion and partial oligoclonal expansion of the infected cells.

## Results

### Sample features and SBH band status

**Figure 1:**
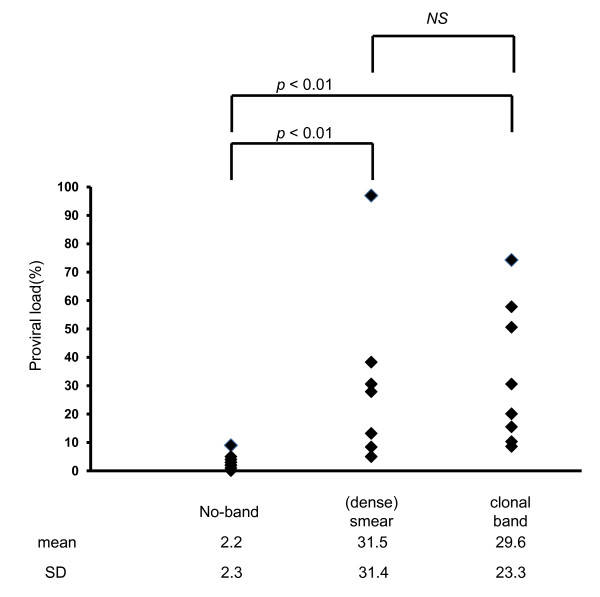
**The distribution of HTLV-1 proviral load (VL) per 100 blood mononuclear cells (MNC) in each sample among the no-band, dense smears and clonal band(s) groups classified according to Southern blotting (SBH) status**. Short bar; median VL.

The median age of the 29 subjects who donated peripheral blood was 66 years old (range, 49-81). No circulating ATL cells were found morphologically or immunophenotyically in any samples, including 8 samples of lymphoma-type ATL employed as a control. Subsequently, all 33 samples were divided into 3 groups; 13 asymptomatic healthy carriers (median age, 60), 12 symptomatic carriers (median age, 68) with complications unrelated to HTLV-1 such as infectious diseases (Strongyloides, hepatic disorders due to HBV and HCV, chronic pneumonitis or bronchitis) and immune-disorders (Crow-Fukase syndrome, RA, and chronic eczema, and reactive unknown adenitis) and 8 patients with lymphoma-type ATL. The distribution of SBH band patterns in each group is summarized in Table 1 and the median proviral loads of the no-band, dense smears and clonal band groups were 2.0% (range, 0.1 - 9.0), 27.9% (5.0-97.4), and 20.1% (8.3-74.3), respectively, as shown in Table 1 and Figure 1.

**Table 1:** The distribution of HTLV-1 SBH band status in samples without circulating ATL cells among three HTLV-1-seropsitive groups, asymptomatic (healthy), symptomatic carriers with HTLV-1-unrelated disorders and patients with lymphoma type ATL.

		SBH			
		
HTLV-1 seropositive persons	No.	no-band*	dense smears	clonal band(s)	total
asymptomatic (healthy)	13	11 (84.6%)	2 (15.4%)	0 (0%)	100%
symptomatic, (complication unrelated to HTLV-1)	12	4 (33.3%)	2 (16.7%)	6(4)**(50.0%)	100%

patients with lym. type ATL***	8	1 (12.5%)	3 (37.5%)	4 (1)**(50.0%)	100%

total	33	16 (48.5%)	7(21.2%)	10 (5)**(30.3%)	100%

*: no-band with or without vague smears

**: aberrant bands showing broad bands

***: patients with lym. Type ATL was defined as cases with no morphological and immunophenotypical abnormal lymphocytes

(): the number of cases with co-existence of clonal band(s) and dense smear bands

### Characteristic band patterns in samples with high VL

**Figure 2:**
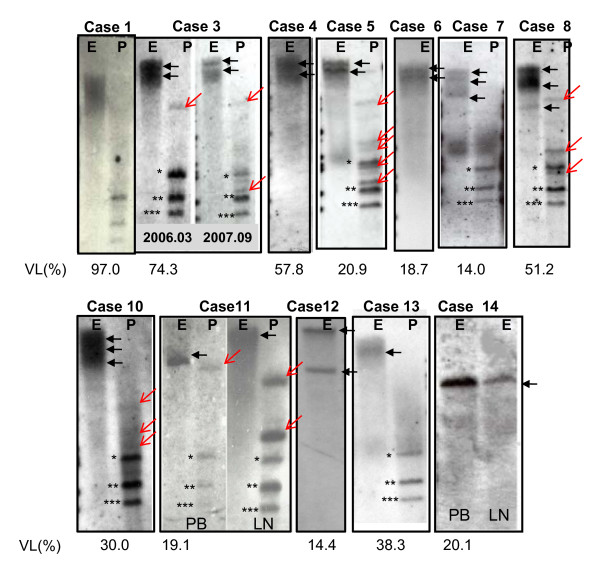
**Representative band patterns by SBH analysis in high VL carriers with dense smears, aberrant-bands with/without faint sharp band(s)**. E & P; EcoR1 and Pst-1 digestion, *, **, ***; internal bands after Pst-1 digestion, ←; clonal band(s), PB; peripheral blood, LN; lymph node. Case 1: Typical dense smeared band, case 3: probably two bands within dense smeared bands in March, 2006, and clear multi-bands in September, 2007. Cases 4, 5, 6, 7, 8, and 10; two or more vague clonal band(s). Case 11; different band sizes between peripheral blood (PB) and lymph nodes (LN), Cases 12 and 13; two clonal bands and an atypical broad band, Case 14; the same band size for LN and blood.

**Figure 3:**
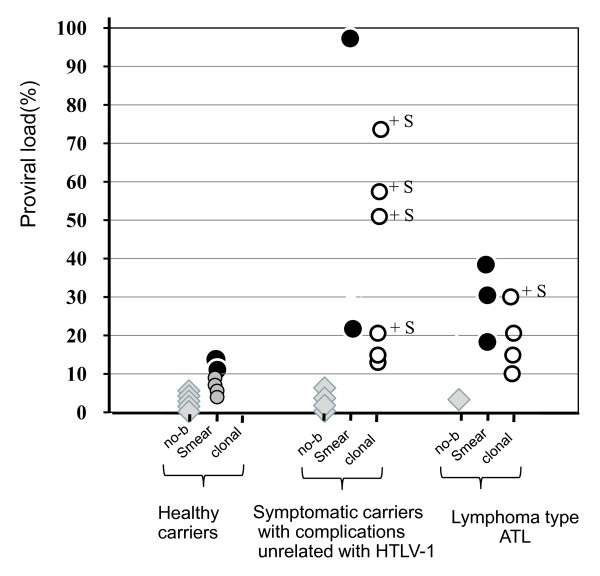
**A comparison between the level of VL and SBH status among healthy asymptomatic carriers, symptomatic carriers with complications unrelated to HTLV-1, and patients with lymphoma type ATL**. Gray diamond; no-band, gray circle; vague to faint smears, solid circle; dense smears, open circle; clonal band, +S: mixture band of dense smears and conal bands.

**Figure 4:**
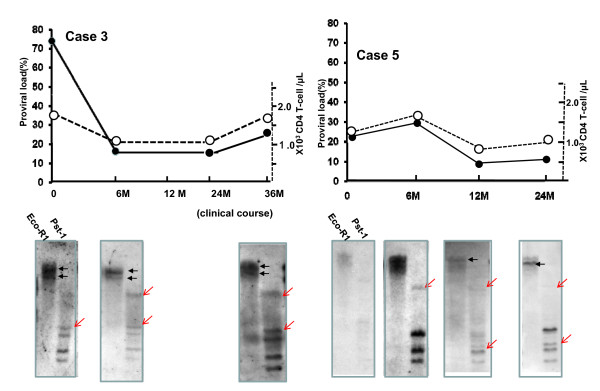
**Upper panel shows the sequential measurement of VL (%; solid line) and CD4T-cell number/μL (broken line) in symptomatic cases 3 and 5**. Lower panel shows SBH status at the same time as VL assays. In case 3, the first SBH gave dense smears (black arrow, Eco-R1 digestion) and an indeterminate clonal band (red arrow, Pst1 digestion), and then dense smears disappeared followed by clear visualization of a clonal band. In case 5, when dense smears emerged, the VL was the highest, and after disappearance, the clonal band and level of VL stably persisted.

Although SBH in asymptomatic carriers gave no clonal band with or without very faint Smears, SBH in some symptomatic carriers gave characteristic band patterns, as shown in Figure 2. Those were mainly a mixture type of dense smears and discrete oligoclonal bands in symptomatic carriers with high VL, such as cases 1 (VL, 97%), 3 (74%), 4 (57%), and 5 (21%). On the other hand, in samples from lymphoma-type ATL, the mixture type was detected in only case 10 and the clonal band type was detected in case 10 to 15. For all sample tested, the relationship between VL and band status is depicted in Figure 3, showing no-band or vague smears in all but one of the healthy carriers, either dense smears or a mixture of dense smears and oligoclonal bands (open circle+S:○+S) in symptomatic carriers and mainly discrete clonal band in patients with lymphoma-type ATL. In particular, an extremely high VL of 50% or more was limited to symptomatic carriers whose band findings always contained at least dense smears. Moreover, as shown in Figure 4, sequential samples disclosed that a higher VL value was synchronized with the transient emergence of dense smears, and declined at the same time as disappearing dense smears. After that, several discrete bands became visible and were persistently retained.

### Clinical features in symptomatic carriers and patients with lymphoma-type ATL

Clinico-hematological features in 15 cases with a high VL of 10% or more and distinctive band patterns are summarized in Additional file 1. Of 8 symptomatic carriers, complicated disorders were mainly associated with abnormal immunity or non-bacterial pathogens. Two of 8 symptomatic carriers developed ATL, case 3 in 4 years later and case 5 in 3.5 years later, respectively. For lymphoma-type ATL, SBH for Lymph node (LN) suspension cells gave positive results in 5 of 6 samples tested. The band size was different in case 11 and was accordant in case 14 between PB and LN, while the other band profiles were very similar to those of symptomatic carriers; they were like a relic of the symptomatic carriers' past.

## Discussion

Recent studies including our previous studies [13,15] suggest that VL in asymptomatic carriers may be approximately one copy per 25 to 1000 MNC. Even in patients with HAM whose VL are known to be high, the VL may be as high as one copy per 10 to 100 MNC. Therefore, we defined a VL of 10% or more per 100 MNC as unusually high.

In the present study, we found that the results of VL and SBH status in healthy carriers were the same as those of the past reports, while there was an extremely high VL with a characteristic band status of high dense smears with or without clonal bands in elderly symptomatic carriers. A VL of 10% or more (range, 10 to 97.4%) was detected in 16 (43.5%) of all samples, 1 (6.3%)/16 asymptomatic healthy carriers, 8 (61.5%)/13 symptomatic carriers unrelated to HTLV-1 and 7(87.5%)/8 patients with lymphoma-type ATL without circulating ATL cells. On the other hand, in SBH analysis, no visible aberrant bands were detectable in low VL samples with less than 10%. All but one of the asymptomatic carriers (mean age; 60) were of this pattern. In contrast, the high VL samples with 10% or more displayed distinctive band patterns accompanied by dense smears with or without discrete clonal band(s), indicating that an increase in polyclonally infected-cells corresponding to dense smears contributed to a high VL. As triggering factors for HTLV-1-infected cells, various microbes and abnormal immunity due to aging in symptomatic carriers were suspected. Furthermore, the observations from sequential samples also support the contribution of dense smears to the elevation of VL. This helps explain the process by which the clonality of HTLV-1-infected cells is established after the disappearance of dense smears.

It is now recognized that clonal expansion of HTLV-1-infected cells is the norm in nonmalignant disease [11]. In the present study, of 13 asymptomatic and 12 symptomatic carriers, the incidence of clonality was 24.0% (6/25 cases), of which 4 cases were accompanied by dense smears and maintained a higher VL. In other word, this suggests that polyclonal expansion, rather than oligoclonal expansion, contributes to a high VL. The contribution of clonal expansion to the elevation of VL in carriers is thought to be small because VL in HAM patients is generally reported to be around 10% on average. In fact, Furukawa et al. [8] reported a high frequency of clonality of 20% in HAM patients and 16% in carriers in families of HAM patients, while the VL was at most 10 to 20% in general. On the other hand, patients co-infected with strongyloidosis and HTLV-1 have been reported to harbor a higher VL of around 50% with a high incidence (39%) of clonality [16,17]. The relation between VL and clonality is controversial [11,12] because the decision regarding clonality depends on the sensitivity of the method.

Taken together, our study supports the idea that extremely high VL mainly results from polyclonal expansion of HTLV-1 infected cells at the sensitive level of SBH.

**Figure 5:**
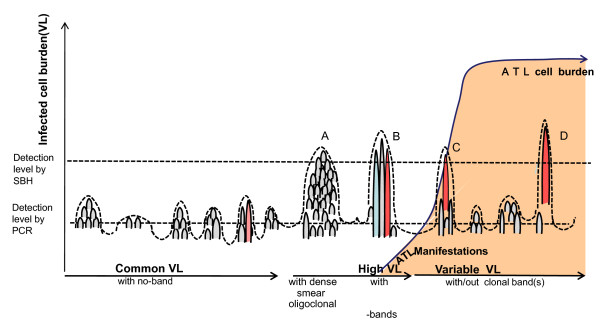
**Conceptual scheme of the fluctuations in the total proviral load (VL) in peripheral blood with no circulating abnormal cells during multi-step leukemogenesis**. VL is low in the early phase of the latent period, during which infected cells consist of clones with a small population. At some time point, some clones may become large enough to be detected as bands by Southern blotting. Peak A corresponds to smears detected as polyclonal expansion by SBH, and then peaks B, C and D appear with oligoclonal bands.

In samples from patients with lymphoma-type ATL without ATL cells, a clonal band(s) was demonstrated in 4 of the 8 patients. Band size between blood and lymph nodes was the same in two of the 4 cases, but no circulating ATL was found. Other aberrant bands, as summarized in Additional file 1, were also observed. Such a band profile in ATL (lymphoma-type) appears to be looks a relic of a symptomatic carriers' past, indicating that high VL with aberrant bands could become a biomarker to predict the development of ATL. Indeed, cases 3 and 5 developed smoldering ATL after 4 years and acute ATL after 3 years, respectively. A conceptual scheme is presented in Figure 5, which shows the biological significance of the fluctuation in a high VL with either dense smears or oligoclonal bands durig multi-step leukomogenesis as a stone corner of dense smear emergence.

## Conclusion

Focusing on not only discrete clonal band(s), but also aberrant smears, it is noteworthy that the emergence of dense smears equivalent to polyclonal expansion of infected cells mainly contributed to a high level VL. However, it is reasonable that a high VL does not consist of only polyclonally expanded infected cells, but included abundant small clones in the cell population, because of sensitivity in SBH. SBH for HTLV-1 could evaluate or monitor the clinical clonal status of HTLV-1-infected cells. Clinically, the distinctive profile of high VL and aberrant band status is expected to monitor or predict some events caused by HTLV-1.

## Materials and methods

### Samples

Samples were collected from our ATL clinical laboratory, consisting of 16 asymptomatic and 13 symptomatic carriers and 8 patients with lymphoma-type ATL as a control because this type of ATL has no circulating ATL cells. A total of 37 samples were seropositive for HTLV-1 and undetectable morphologically and immunophenotypically for ATL cells, indicating all samples had no evidence of circulating ATL cells.

### Serologic and genomic assays for HTLV-1

Anti-HTLV-1 antibodies were detected by chemiluminescent enzyme immunoassay (Fuji Rebio, Tokyo, Japan). High-molecular-weight DNA was extracted from blood mononuclear cells (MNCs) using a QIAmp DNA Blood Mini kit (Qiagen GmbH, Hilden, Germany). VL was quantified by LightCycler Technology (Roche Diagnostics KK, Tokyo, Japan) using hydro-probes and previously described primers [15], with β-globin as an internal control. The PCR methodology was monitored by determining the amount of β-globin DNA required to generate 10,000 copies per 5,000 MNC. We assumed that one infected cell harbored one provirus, and the number of infected cells was therefore estimated to be the same as the proviral copy number and was expressed per 100 MNC (% or load).

### Clonal assay by SBH

SBH analysis was performed as described previously [18,19], using mixture probes covering the total region of the provirus digoxigenined and the restriction enzymes *EcoR1 *and *Pst-1*. There are four *Pst-1 *sites but no *EcoR1 *site within the proviral sequence. Accordingly, if *EcoR1*-digestion gave no-band or faint smears and *Pst-1*-digestion gave only three internal bands, the sample was considered to be negative for clonal expansion. When discernible discrete band(s) in the *EcoR1*-digestion membrane or one or two external band(s) in addition to three internal bands in the *Pst-1*-digestion membrane were visible, the sample was considered to harbor clonal integrated provirus, implying that the infected cells had expanded clonally. The results of SBH analysis were classified into three patterns; no-band, dense smears and one or more discrete band(s). "Dense" smears were assumed to be distinct from those of common carriers (3 fold< smear density relative to background lane densitiy). The detection sensitivity for clonally infected cells in the SBH assay was 3-5% [18]. The sensitivity was monitored in each blotting membrane using 3-5% clonal cells from the ST1 ATL cell line.

### Statistics

Data were analyzed using Mann-Whitney or Chi-squared tests. Statistical significance was set at p < 0.05.

## Abbreviations

HTLV-1: human T-cell leukemia virus type-1; ATL: adult T-cell Leukemia; VL: HTLV-1 proviral load; SBH: Southern blot hybridization; PCR: polymerase chain reaction.

## Competing interests

The authors declare that they have no competing interests.

## Authors' contributions

SK, DS, and TW conceived this study and provided funding. DS, HH, KY, YY and KT collected samples and carried out the molecular genetic studies. MI, TW, AO and SK analyzed the data and discussed the results. SK organized the study and wrote the manuscript. All authors read and approved the final manuscript.

## Supplementary Material

Additional file 1 **Summary of the main clinical and laboratory data in seropsitive individuals with high VL and aberrant band patterns in SBH, and outcome in Dec 2008. Two cases (#3 and 5) among 8 advanced carriers (cases 1 to 8) developed ATL 4 and 3 years later**. Cases 1, 2 and 15: High VL carriers with polyclonal expansion. Cases 3-14; aberrant bands mainly with faint multiple clonal bands, Final diagnosis was based on the integrated findings of an LN SBH test and clinico-pathological examinations. ALCL; anaplastic large cell lymphoma, DLBCL; diffuse large cell B-cell lymphoma, (-) or (+); negative or positive clonal band, S; smear, B; band, NT: not tested, Dx: diagnosis, *: indeterminate for clonal band, **: pathological diagnosis was indeterminate. For the other abbreviations; refer to the context. Click here for file
