# Supplementary material for: High Human T Cell Leukemia Virus Type-1(HTLV-1) Provirus Load in Patients with HTLV-1 Carriers Complicated with HTLV-1-unrelated disorders
Source: Virol J. 2010 Apr 28;7:81. doi: 10.1186/1743-422X-7-81 (PMC2876101; doi:10.1186/1743-422X-7-81)
Supplement: Additional file 1 — Summary of the main clinical and laboratory data in seropsitive individuals with high VL and aberrant band patterns in SBH, and outcome in Dec 2008. Two cases (#3 and 5) among 8 advanced carriers (cases 1 to 8) developed ATL 4 and 3 years later. Cases 1, 2 and 15: High VL carriers with polyclonal expansion. Cases 3-14; aberrant bands mainly with faint multiple clonal bands, Final diagnosis was based on the integrated findings of an LN SBH test and clinico-pathological examinations. ALCL; anaplastic large cell lymphoma, DLBCL; diffuse large cell B-cell lymphoma, (-) or (+); negative or positive clonal band, S; smear, B; band, NT: not tested, Dx: diagnosis, *: indeterminate for clonal band, **: pathological diagnosis was indeterminate. For the other abbreviations; refer to the context. [file 1743-422X-7-81-S1.PPT]

## Slide 1
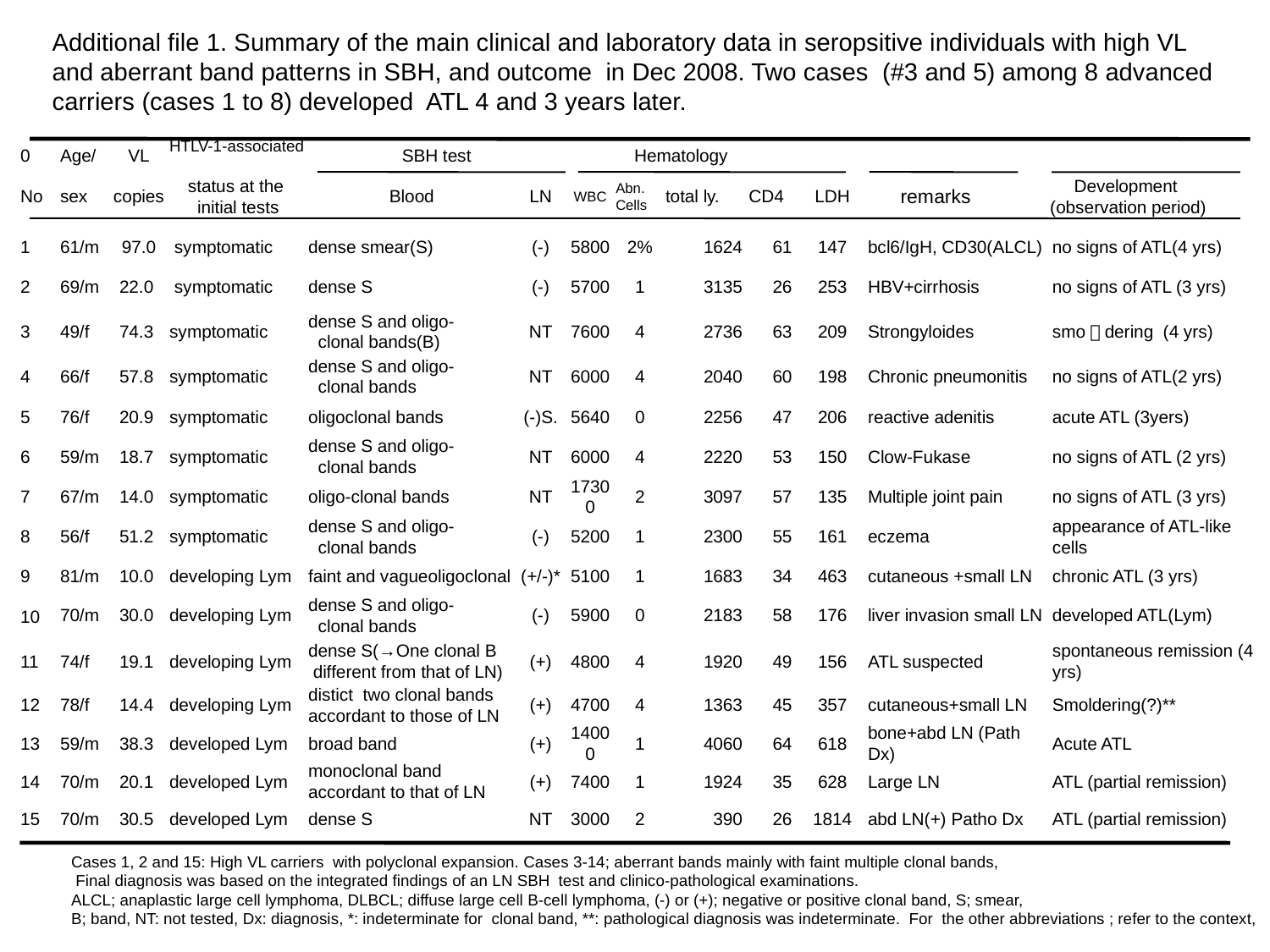

Additional file 1. Summary of the main clinical and laboratory data in seropsitive individuals with high VL and aberrant band patterns in SBH, and outcome in Dec 2008. Two cases (#3 and 5) among 8 advanced carriers (cases 1 to 8) developed ATL 4 and 3 years later.
0
Age/
VL
HTLV-1-associated
SBH test
 Hematology
No
sex
copies
status at the initial tests
Blood
LN
WBC
Abn.
Cells
total ly.
CD4
LDH
remarks
Development
(observation period)
1
61/m
97.0
 symptomatic
dense smear(S)
(-)
5800
2%
1624
61
147
bcl6/IgH, CD30(ALCL)
no signs of ATL(4 yrs)
2
69/m
22.0
 symptomatic
dense S
(-)
5700
1
3135
26
253
HBV+cirrhosis
no signs of ATL (3 yrs)
3
49/f
74.3
symptomatic
dense S and oligo-
 clonal bands(B)
NT
7600
4
2736
63
209
Strongyloides
smoｌdering (4 yrs)
4
66/f
57.8
symptomatic
dense S and oligo-
 clonal bands
NT
6000
4
2040
60
198
Chronic pneumonitis
no signs of ATL(2 yrs)
5
76/f
20.9
symptomatic
oligoclonal bands
(-)S.
5640
0
2256
47
206
reactive adenitis
acute ATL (3yers)
6
59/m
18.7
symptomatic
dense S and oligo-
 clonal bands
NT
6000
4
2220
53
150
Clow-Fukase
no signs of ATL (2 yrs)
7
67/m
14.0
symptomatic
oligo-clonal bands
NT
17300
2
3097
57
135
Multiple joint pain
no signs of ATL (3 yrs)
8
56/f
51.2
symptomatic
dense S and oligo-
 clonal bands
(-)
5200
1
2300
55
161
eczema
appearance of ATL-like cells
9
81/m
10.0
developing Lym
faint and vagueoligoclonal
(+/-)*
5100
1
1683
34
463
cutaneous +small LN
chronic ATL (3 yrs)
70/m
30.0
developing Lym
dense S and oligo-
 clonal bands
(-)
5900
0
2183
58
176
liver invasion small LN
developed ATL(Lym)
10
11
74/f
19.1
developing Lym
dense S(→One clonal B
 different from that of LN)
(+)
4800
4
1920
49
156
ATL suspected
spontaneous remission (4 yrs)
12
78/f
14.4
developing Lym
distict two clonal bands
accordant to those of LN
(+)
4700
4
1363
45
357
cutaneous+small LN
Smoldering(?)**
13
59/m
38.3
developed Lym
broad band
(+)
14000
1
4060
64
618
bone+abd LN (Path Dx)
Acute ATL
14
70/m
20.1
developed Lym
monoclonal band accordant to that of LN
(+)
7400
1
1924
35
628
Large LN
ATL (partial remission)
15
70/m
30.5
developed Lym
dense S
NT
3000
2
390
26
1814
abd LN(+) Patho Dx
ATL (partial remission)
Cases 1, 2 and 15: High VL carriers with polyclonal expansion. Cases 3-14; aberrant bands mainly with faint multiple clonal bands,
 Final diagnosis was based on the integrated findings of an LN SBH test and clinico-pathological examinations.
ALCL; anaplastic large cell lymphoma, DLBCL; diffuse large cell B-cell lymphoma, (-) or (+); negative or positive clonal band, S; smear,
B; band, NT: not tested, Dx: diagnosis, *: indeterminate for clonal band, **: pathological diagnosis was indeterminate. For the other abbreviations ; refer to the context,
